# Hierarchical Biobased Macroporous/Mesoporous Carbon: Fabrication, Characterization and Electrochemical/Ion Exchange Properties

**DOI:** 10.3390/ma16052101

**Published:** 2023-03-05

**Authors:** Mariano M. Bruno, N. Gustavo Cotella, Cesar A. Barbero

**Affiliations:** Research Institute for Energy Technologies and Advanced Materials (IITEMA), National University of Río Cuarto (UNRC)-National Council of Scientific and Technical Research (CONICET), Río Cuarto 5800, Argentina

**Keywords:** hierarchical, mesoporous carbon, cellulosic, fabric, supercapacitor

## Abstract

With the goal of improving the mechanical properties of porous hierarchical carbon, cellulosic fiber fabric was incorporated into the resorcinol/formaldehyde (RF) precursor resins. The composites were carbonized in an inert atmosphere, and the carbonization process was monitored by TGA/MS. The mechanical properties, evaluated by nanoindentation, show an increase in the elastic modulus due to the reinforcing effect of the carbonized fiber fabric. It was found that the adsorption of the RF resin precursor onto the fabric stabilizes its porosity (micro and mesopores) during drying while incorporating macropores. The textural properties are evaluated by N_2_ adsorption isotherm, which shows a surface area (BET) of 558 m^2^g^−1^. The electrochemical properties of the porous carbon are evaluated by cyclic voltammetry (CV), chronocoulometry (CC), and electrochemical impedance spectroscopy (EIS). Specific capacitances (in 1 M H_2_SO_4_) of up to 182 Fg^−1^ (CV) and 160 Fg^−1^ (EIS) are measured. The potential-driven ion exchange was evaluated using Probe Bean Deflection techniques. It is observed that ions (protons) are expulsed upon oxidation in acid media by the oxidation of hydroquinone moieties present on the carbon surface. In neutral media, when the potential is varied from values negative to positive of the potential of zero charge, cation release, followed by anion insertion, is found.

## 1. Introduction

Porous carbon materials can be applied in a variety of fields, including electrochemical energy storage [1,2,3,4,5,6,7,8,9], gas adsorption [10,11], catalysis [12,13,14], catalyst support [15,16,17,18], water treatment [19,20,21,22,23,24,25,26], decontamination [27,28,29], absorption of volatiles organic contaminants (VOC) [30,31], and photothermal heating [32], etc. In most of those applications, large surface areas with good accessibility are required. Hierarchical materials with macro- and meso/microporosity show medium-large surface areas (meso/microporosity) and good mass transport of species from the surrounding media (macroporosity) [33]. For the adsorption of species from the solution, the solvent should be able to wet the whole surface area. Hierarchical pore structures with a distribution of macro, meso, and micropores produce easy accessibility, fast ionic moiety, and high surface area with a short pore length, addressing the issue of carbon with monomodal nanopore structure.

Due to the high surface tension of water, mesopores can mostly be wet in aqueous solutions, and porous carbon with large mesoporosity presents larger exposed areas than microporous carbons [34].

Several methods can be used to produce porous carbon materials [35]. The conventional way to produce porous carbon from biomass is the carbonization of lignocellulosic biomass (e.g., wood) to produce carbons, followed by activation to produce high surface area materials [36]. The process renders large surface area materials, but usually with a significant degree of microporosity. Those materials are useful for the adsorption of volatile molecules, but a large part of the surface area is not wetted by liquids (e.g., water). On the other hand, there has been extensive work in the production of synthetic mesoporous carbons based on resorcinol/formaldehyde (RF) chemistry [37]. Hard and soft templating methods were used to produce porous precursor RF dry gels [38,39]. RF gels are intrinsically porous, but the evaporation of water in air produces non-porous xerogels [40]. Water, and other solvents with hydrogen bonding, like alcohols, present high surface tension, which forms a gas/liquid interface (meniscus) inside the pores. Upon evaporation, the surface tension collapses the soft pores of the RF gel. Therefore, diverse solvent removal methods have been used to avoid pore collapse. Pekala and coworkers [37] pioneered the use of supercritical liquid CO_2_ to replace the water inside the pores and maintain the porous structure during drying [41]. Since water is immiscible with liquid CO_2_, water is exchanged by an intermediate solvent (e.g., acetone) which is both miscible with water and liquid CO_2_. Cryogels are also made by fast freezing the wet gel and then sublimating the solid solvent [42]. Since the expansion of solid water (ice) would break the gel, an intermediate solvent (e.g., *t*-butanol) is used to replace the water inside the pores. Finally, ambigels are made by evaporation at ambient pressure of a non-hydrogen bonded solvent (e.g., n-pentane) [43]. Since such solvent is not miscible with the synthesis (water), one or more intermediate solvents must be used for the exchange.

An alternative method involves stabilizing or templating the pore structure. It has been reported that cationic surfactant micelles [44,45,46,47,48,49,50,51], or cationic polyelectrolytes [52,53,54], can stabilize the gel nanoparticles and maintain the porosity of RF gels during air drying. The porous carbons are produced by pyrolysis of the RF gels in an inert atmosphere [55]. The carbons present large surface areas (>500 m^2^ g^−1^) without further activation. The materials, having large surface area and good electrical conductivity, can be applied in electrodes for supercapacitors [56] and as functional catalysts in esterification [57] and transesterification (biodiesel synthesis [58]). Mesoporous inorganic matrices could also be used to template the pores in the resin [59,60]. Porous carbons can be obtained as a powder or in monolithic pieces. However, in the aforementioned applications, monolithic materials offer useful characteristics such as hierarchical pore structure, good electrical conductivity, or easy remotion. Regards monolith material, obtention carbon cloth can be used as porous carbon support. This has demonstrated that it improves the mechanical properties of composite material [61]. But also, the microstructures of fibers have been used as hard templates [62,63]. Similar materials have been produced by the adsorption of RF resin on cellulosic materials but by adding templates [64]. Moreover, it has been reported that a carbon-carbon composite is produced by the carbonization of resorcinol-formaldehyde resins, which are adsorbed onto a cellulosic fiber fabric [65].

Cellulosic fibers can be used to reinforce composites mechanically [66,67,68]. Therefore, the original goal was to reinforce the RF resin using the fiber cloth. Self-sustaining porous carbons have been produced by adsorbing RF resin on cellulosic materials but by adding templates as pore-forming. However, cellulosic fiber fabric has been demonstrated to act as a structuring agent for resorcinol-formaldehyde resins to produce carbon-carbon composites [65]. The method produces a carbon with nanometric-sized pores (meso and micropores) and micrometric-sized pores (macropores), making it a hierarchical structure. While the nanopores give the material a large surface area which could be used to store electrical charge in an electrochemical system, the macropores allow fast access to the electrolyte, making the charge storage faster. Moreover, the hierarchical structure could also be beneficial for gas adsorption under forced flux [69]. Moreover, the material is easily made as monoliths in a simple process, an important goal for the application of carbon materials [70]. It has been suggested that hierarchical carbons have the advantage over monotonic porous materials in their application for electrochemical sensors [71] and electrodes for fuel cells [72]. Physical sensors (pressure, movement) have been built using carbonized cellulosic (cotton) fabric alone [73,74,75] or decorated with graphene [76]. Cellulosic fibers [77], including cotton [78], have been activated to produce high surface area electrode materials for supercapacitors. Du et al. adsorb a metal-organic framework (ZIF) to create a large surface area of doped N-carbon with the same goal [79].

In the present work, a detailed study of the fiber stabilization method to produce hierarchical (macro/meso/microporous) carbon is carried out. The electrochemical and potential-driven ion exchange properties are also studied. To the best of our knowledge, this is the first study that shows both improvements in the mechanical properties and stabilization of the porosity of the RF resin due to the presence of cellulosic fibers. Moreover, it is shown that carbon fibers, obtained by pre-carbonization of the cellulosic fiber cloths, also stabilize the porosity of the resin during air drying, suggesting that the porosity stabilization by fibers is a general method. Therefore, it could be induced by other organic (e.g., carbon nanotubes) or inorganic (e.g., alumina) fiber mats. This way, a one-step method is designed to obtain mechanically stable and hierarchically porous carbon.

## 2. Materials and Methods

All electrochemical experiments were carried out in degassed solutions by extensive N_2_ bubbling. Resorcinol (Aldrich, St. Louis, MO, USA, >99.0%) and formaldehyde (Cicarelli, ACS reagent, 37% with 10–15% methanol as stabilizer) were used as reactants without further purification. Sodium carbonate (decahydrate, Aldrich, >99.0%) and cetyl,trimethylammonium bromide (CTAB, Aldrich, >98%) were used as catalyst and stabilizer, respectively. All other chemicals used were of analytical grade, and solutions were prepared with Millipore water. The cellulosic woven fiber fabric is a raw canvas made of crude cotton. The canvas is washed with detergent to remove any additive of the weaving process and then washed with 1% NaOH solution (48 h with stirring) to remove any impurities. Cellophane film was acquired from a local paper retailer and washed with 1% NaOH overnight to remove any impurities.

### 2.1. Resin Synthesis

The viscous prepolymer solution (Resorcinol (R): Formaldehyde(F):Catalyst(Na_2_CO_3_ (C))) = 1:2.9:0.005) solution was impregnated onto the cellulosic fabric until saturation, while the fabric was stretched in a wood frame. To cure the resin, the materials are left for 24 h at 70 °C inside a closed glass chamber filled with water saturated atmosphere. Then, the resins were dried in an open oven at 70 °C for 24 h. The weight loss during drying is ca. 45% in weight. The composite consists of an RF resin supported on a woven cellulosic fiber fabric (RF@F).

To produce the composite on a regenerated cellulose film (cellophane) (RF@cp), a thin layer of prepolymer solution was painted on the film’s surface and processed in the same way.

To produce the mesoporous resin using a surfactant as a stabilizer of the porosity (RF_CTAB_) [49], enough cetyl,trimethylammonium bromide (CTAB) to the polymerization solution to set a molar ratio of CTAB:resorcinol of 0.06:1. The gels were cured inside close Petri dishes. To produce non-porous RF (for comparison), the same mixture (without CTAB) was placed on the petri dishes.

### 2.2. Carbonization

The carbonization of the samples was carried out by carbonization in an electric oven (5 kW). The heating program was adjusted via a LabView script (National Inst.) controlling a PC-1200 AD/DA card. The card reads the temperature of a “K” thermocouple and commands an electronic relay that controls the electric power input to the oven. The samples were set in a ceramic recipient. The resin layers were separated with ceramic macroporous thick (>5 mm) separators, which hold the resin in place, avoiding deformation. On the other hand, the pyrolysis gases could leave the plates through the separators. A nitrogen flow of 0.2 L min^−1^ was used to maintain the inert atmosphere. The free spaces in the ceramic recipient were filled with purified wood charcoal to act as a sacrificial material in case of oxygen or water contamination. The heating rate used is 40 °C/h from ambient to the pyrolysis temperature. The dry resins are carbonized under a nitrogen stream at 800 °C. The same temperature program was used for all porous carbons produced. The non-porous materials (RF and RF@cp) were carbonized using a slower heating rate (10 °C min^−1^) to allow gas release without breaking. The carbonized materials are denominated c(material) (e.g., c(RF@F)).

### 2.3. Measurement of Surface Area

Porosimetry analysis was done with the Micromeritics ASAP 2000 equipment using N_2_ at −196.15 °C. The specific surface area was calculated by the Brunauer, Emmett, and Teller (BET) method, while the pore size distribution was obtained by the Barrett-Joyner-Halenda (BJH) method from the desorption isotherms.

### 2.4. Scanning Electron Microscopy

Images of carbon materials were made with a scanning electron microscope (SEM) Philips 515. Transverse morphology was obtained by fracturing the samples.

### 2.5. Thermal Analysis during Carbonization

Thermogravimetric (TG) analysis was carried out with a TGA 2050 (TA-Instruments, USA). Moreover, volatile products produced during thermal degradation were analyzed by mass spectrometry (MS). For this purpose, a thermogravimetric analyzer was coupled to the mass spectrometer (Thermostar, Balzers, Liechtenstein). Thermal analyses were made using samples of about 10 mg with a flow of air or nitrogen stream of 165 mL/min gas stream and a heating rate of 10 °C min^−1^.

### 2.6. Measurement of Mechanical Properties

The nanoindentation measurements were carried out with Nanoindenter XP equipment (MTS Systems, Eden Prairie, MN, USA), with a diamond Berkovich indenter (three-sided pyramidal). All measurements were performed at a penetration rate of 50 nm s^−1^ with a maximum penetration rate of 100 nm. The HIT hardness and EIT modulus calculations were carried out according to ISO 14577 standards, and the projected contact area was calculated according to the Oliver and Pharr method [80]. The hardness and modulus measurements depend on three experimentally measurable values of the F-*h* curve: the maximum load, *P*_max_, the maximum displacement, *h*_max_, and the elastic shock stiffness or contact stiffness, S, which is calculated from the slope in the upper portion of the discharge curve. The machine’s rigidity and contact stiffness intervene in this parameter. For convenience, the inverse of S is used (compliance *C*), which has two components: *C_f_*, related to the machine, and *C_s_*, related to the test sample. Modeled as two springs in a series, their relationship is

(1) $$\frac{\partial h}{\partial P}=\frac{1}{S}=C_{f}+C_{s}$$

With this value, the contact depth is calculated:

(2) $$h_{c}=h_{\max}-0.75\frac{P_{\max}}{S}$$

If the geometry of the indenter is a three-sided pyramid, the contact area is calculated:

(3) $$A_{c}=24.5h_{c}{}^{2}$$

Knowing the contact area, the hardness of the material can be calculated:

(4) $$H=\frac{P_{\max}}{A_{c}}$$

To calculate the modulus of elasticity of the material measured by nanoindentation, *E_IT_*, the following Equation is used:

(5) $$\frac{1}{E^{*}}={\left[\frac{1-\rho^{2}{}_{ind}}{E}\right]}_{indenter}+{\left[\frac{1-\rho_{mat}{}^{2}}{E_{IT}}\right]}_{material}$$

called the reduced modulus of elasticity E*, where *ρ* is the Poisson’s ratio of the material and the indenter, respectively. For an indenter of the Berkovich type (three-sided pyramidal) of diamond, the Poisson’s ratio is *ρ*: 0.07, and its module (*E*) is 1141 GPa. In soft materials such as carbon, this term is comparatively negligible to the modulus of elasticity of the sample. Through the experimental determination of the stiffness *S*, the value of the reduced modulus of elasticity can be calculated through the Equation

(6) $$E^{*}=\frac{\sqrt{\pi }}{2}S\frac{1}{\sqrt{A_{c}}}$$

Using E*, it is possible to calculate the material’s indentation modulus, EIT, by solving it from Equation (5).

For the nanoindentation measurements, the samples were supported on a polymeric resin, and its surface was polished to eliminate all existing imperfections which could generate erroneous measurements in the material.

### 2.7. Electrochemical Properties

#### 2.7.1. Cyclic Voltammetry (CV) and Electrochemical Impedance Spectroscopy(EIS)

The electrochemical characterization was carried out in a three-electrode cell using a computerized potentiostat (GAMRY PC4) and CM 300 impedance software. Pieces of carbon aerogel with a geometrical area 5 times the working electrode were used as the counter electrode (CE) while a saturated calomel electrode (SCE) with a saturated NaCl inner solution) was used as the reference electrode (RE). The Electrochemical impedance spectroscopy (EIS) measurements were assessed using a 1 mV voltage amplitude of perturbation of 5 mV and scanning frequencies from 50 kHz to 2.8 mHz. Aerogel (Maketech) pieces were used as counter electrodes. They consist of thin films (ca. 500 μm thickness) with macropores. The BET surface area (measured by nitrogen adsorption) of the aerogels is 700 m^2^g^−1^. This larger piece of porous carbon as a counter electrode allows a low current density, which is the precondition for correct electrochemical measurements.

To calculate the specific capacitance (Sp_cap_ in Fg^−1^) from the voltametric current, Equation (7) is used:

(7) $${\mathrm{Sp}}_{\mathrm{cap}}=\frac{i}{v\times m}$$

where *i* is the voltametric current (A), v is the scan rate (V/s), and m is the mass of the electrode (g).

The cyclic voltametric responses of a simple RC circuit were simulated (see Figures S6 and S7 in the Supplementary Materials) using the equations described by Bard and Faulkner [81]. The C corresponds to the double-layer capacitance, and the R is composed of the resistances in series, including the electrical resistance of the carbon electrode, the resistance of the solution, and the mass transports inside the carbon. The calculations were made in Excell 2010 and plotted in Origin 9.

#### 2.7.2. Chronoamperometry (CA)

The CA measurements were made simultaneously with the PBD measurement using an AMEL 2049 Potentiostat controlled by a LabPC AD/DA card under Labview. The chronoamperometry profiles (*i*-t) were integrated using Origin 9 to obtain the electrochemical charge.

#### 2.7.3. Probe Beam Deflection

Probe Beam Deflection detects the concentration gradient in front of the electrode by measuring the refractive index gradient with a light beam. The beam travels parallel to the surface and suffers a deviation proportional to the concentration gradient. Therefore, it is related to the amount and direction of ion flux. Negative beam deflection (towards the electrode) corresponds to the release of ions from the electrode to the solution, while positive deflection (from the electrode) implies the intake of ions into the electrode. The Probe Beam Deflection arrangement was similar to the one described before [82]. The experimental details are described in the Supplementary Materials.

## 3. Results and Discussion

### 3.1. Fabrication of RF Carbon Supported on Cellulosic Woven Fabric

**Scheme 1:**
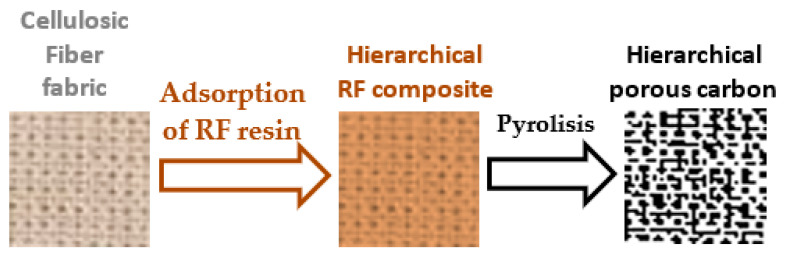
Description of the different steps used to produce a hierarchical porous carbon.

The viscous prepolymer solution was impregnated onto the cellulosic fabric), until saturation while the fabric was stretched in a wood frame. Then, they are cured, dried, and carbonized (Scheme 1).

**Figure 1:**
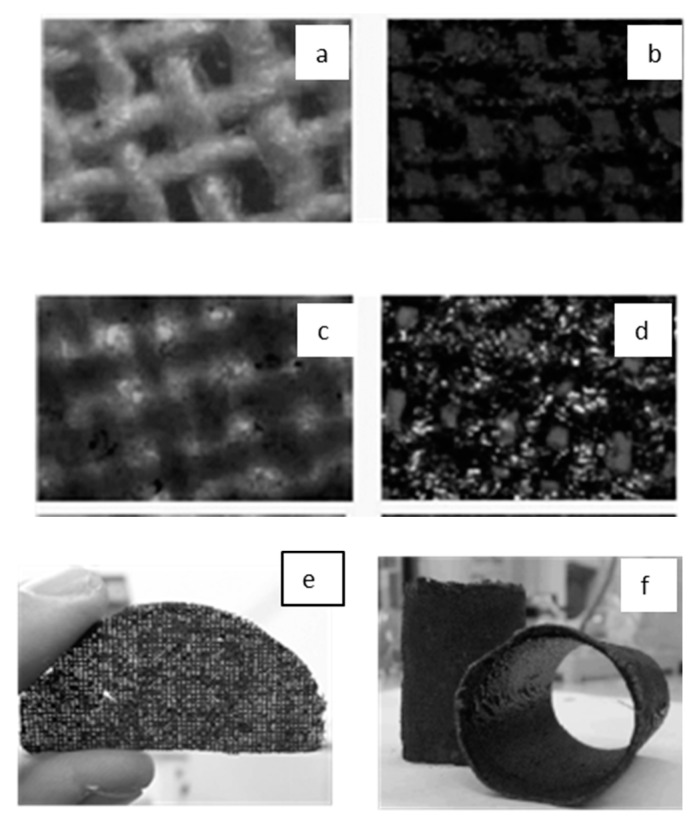
(**a**–**d**) Optical micrographs (60×) of (**a**) woven cellulose fiber fabric; (**b**) carbonized fabric (**a**); (**c**) fabric with adsorbed resin; (**d**) carbonized composite (**c**); (**e**,**f**) Photographs (1×) of: (**e**) one layer of fiber fabric with adsorbed RF resin; (**f**) cylinders made by attaching four layers of fiber fabric with adsorbed RF resin.

The woven cellulosic fiber carbonizes, forming a carbon cloth with shrinking but retaining its macropores without melting (Figure 1a,b). This is the typical behavior of carbonized cellulose [83]. Visually, it can be seen that the resin decorates the cellulosic fiber (Figure 1c,d), and the carbon deposit maintains the macroporous woven structure with a non-homogenous deposit of RF, with contraction [84], due to the release of polymer fragments (see Section 3.3.3). The macropores allow the release of the decomposition gases without breaking the material, as happens with monolithic carbons [85]. Therefore, fast heating rates can be used. Since RF resins are widely used as adhesives [86], it is then possible to create multilayer assemblies by adhesion of individual fabric layers. Using the procedure described in the experimental part, complex shapes, multilayers, and extensive (up to 15 cm dia.) pieces could be easily assembled by the method (Figure 1e,f).

### 3.2. Mechanical Properties

**Figure 2:**
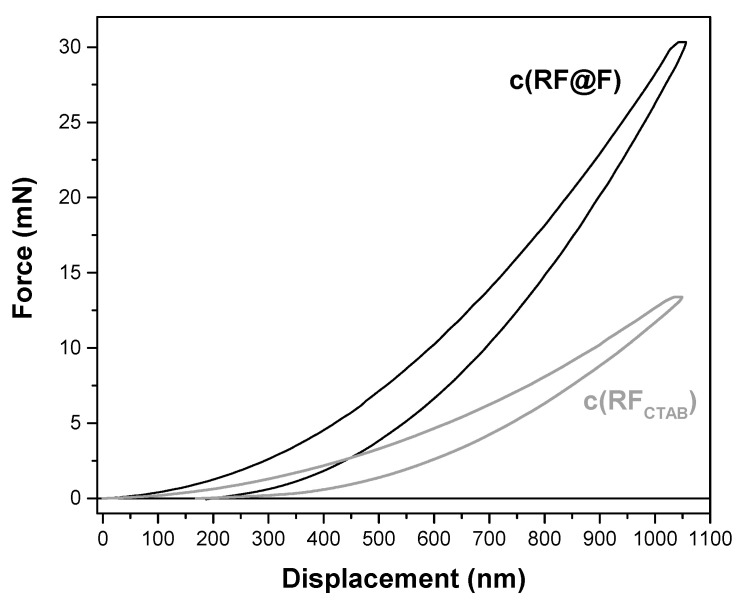
Nanoindentation profiles of hierarchical c(RF@F) (black line) and mesoporous c(RF_CTAB_) (gray line).

The initial objective of compositing an RF resin with a woven cellulosic fiber was the improvement of the mechanical properties (e.g., elasticity) since glassy carbon show high hardness but low elasticity [87]. To test the mechanical properties, nanoindentation was used. In the case of porous materials, the properties will also depend on the material’s porosity. Since indentation resistance increases with decreasing porosity, hardness may increase with the depth of penetration as a result of the densification of the material [88]. Figure 2 shows the load curves versus displacement (F-h) of composite (c(RF@F)) and carbonized mesoporous carbon (c(RF_CTAB_)). The displacement force behavior exhibits almost complete recovery with some hysteresis between charge and discharge. This is elastic-plastic behavior that, in some cases, can produce residual penetration caused by irreversible plastic deformation of the carbon. They all present a similar behavior where the residual plastic deformation is approx. 300 nm. These results would indicate that, under the conditions used during the tests, the densification of materials with a high specific surface area is not due to their porosity.

Qualitatively, it can be seen that the carbon made by pyrolysis of mesoporous RF reinforced with cellulosic fiber is more elastic than the carbon produced from mesoporous RF (RF_CTAB_). Using the Oliver and Pharr model (Equations (5) and (6) of the experimental part) [80], it is possible to calculate the values of the elastic modulus and hardness (Table 1). In this case, the contact area was calculated, proposing an elastic model in which material pile-up is negligible [80].

The calculated values confirm that the elastic modulus of the composite is larger than that of the porous, not reinforced carbon. This is reasonable as the carbon fiber matrix acts as reinforcement. The hardness of the material is also ca. two times larger. This is surprising since the material contains macropores (see Section 3.1) which should reduce the overall resistance. However, it seems that the presence of carbon fiber fabric from cellulose carbonization increases resistance at the nanometric level. The improved mechanical properties are relevant for technological applications of the materials.

### 3.3. Morphology and Textural Properties

Since it was apparent that mechanically stable composites (carbon fabric/RF carbon) could be produced, the nanoparticle stabilizing method was applied to add CTAB to the polymerization mixture. As a control, the sample without CTAB was also tested. Surprisingly, both samples show similar textural properties.

#### 3.3.1. Morphology

**Figure 3:**
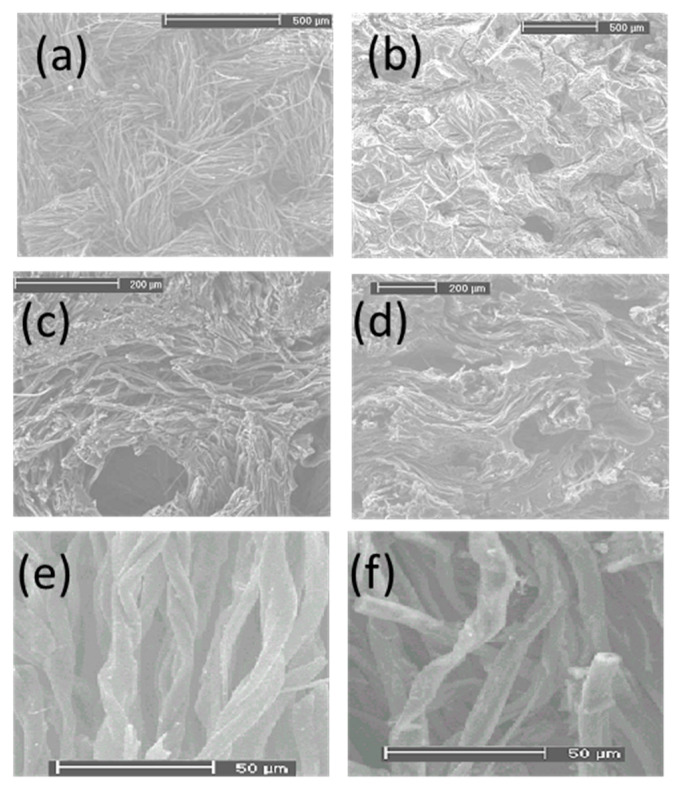
SEM micrographs of the carbonized materials: (**a**) carbonized fabric (**b**,**c**) carbonized composite (surface); (**d**) carbonized composite (fracture), (**e**) carbonized fabric, (**f**) carbonized c(RF@F) (surface). The macropores arise from the holes in the fabric and the space between fibers. Therefore, even if a macroscopically compact fabric is used (e.g., non-woven fabric), the space between fibers would act as macropores.

Figure 3 are shown the SEM micrographs of the carbonized fabric, which was treated with RF, compared to the carbonized fabric. High-resolution images of carbonized fabric look quite similar (Figure 3e) to the cellulosic fabric carbonized in the same conditions (Figure 3f), suggesting that the fabric fiber morphology is maintained the sol-gel, as it has been shown to occur with titania [89].

#### 3.3.2. Textural Properties

**Figure 4:**
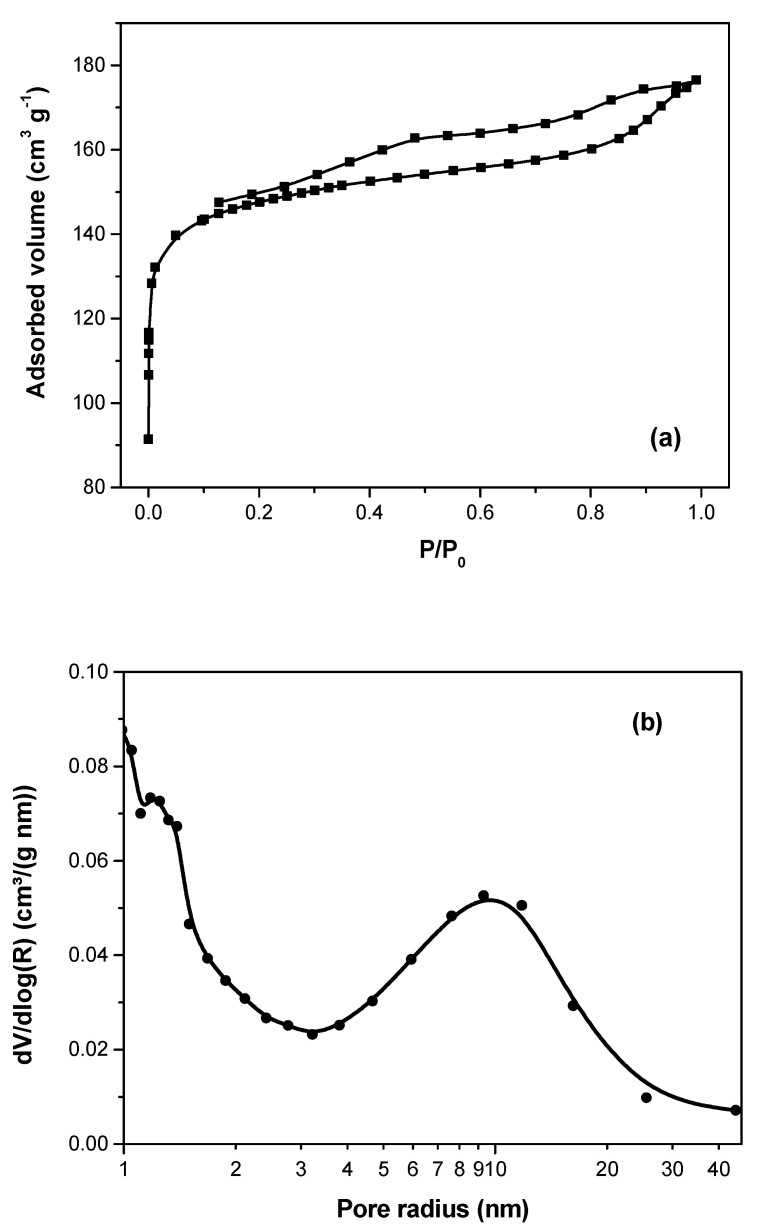
N_2_ adsorption/desorption isotherm (**a, balck squares**) and pore size distribution (**b, black dots**) of c(RF@F). The results show that natural fiber with resorcinol-formaldehyde composite forms hierarchical carbon pore structures from different scaffolds. Micrometric millipore allows easy access into the monolith material and bimodal mesopores and micropores that contributes to obtaining a high surface area but remains good accessibility to the nanometric scale. The values are lower than those obtained for activated carbon from cotton (>1000 m^2^ g^−1^ [91]), but the large mesoporosity allows electrolyte penetration and large specific capacitances (see Section 3.3.4).

Figure 4a shows the nitrogen adsorption-desorption isotherms. At low pressure, the adsorption profile shows a high increase which is characteristic of micropore presence, but also, the hysteresis loop formed by the adsorption-desorption isotherms is indicative of mesopore existence. The loop can be classified as Type H4, which is attributed to slit-like pores [90].

Moreover, it can be observed that the hysteresis loop remains open at low pressure. This unusual behavior can be attributed to the natural fiber as a pore former. This produces micropores and a broad range of mesopores. Therefore, a plausible explanation is some pores are not accessible to the N_2_ gas, which leads to incomplete filling at low relative pressures. A value of 558 m^2^g^−1^ of specific surface area was calculated from the BET method.

Figure 4b shows the pore size distribution calculated using the BJH method. A bimodal pores size distribution can be observed. Large mesopores (10 mm in radius) and a lower size (below 2 nm in radius).

#### 3.3.3. Carbonization Process

**Figure 5:**
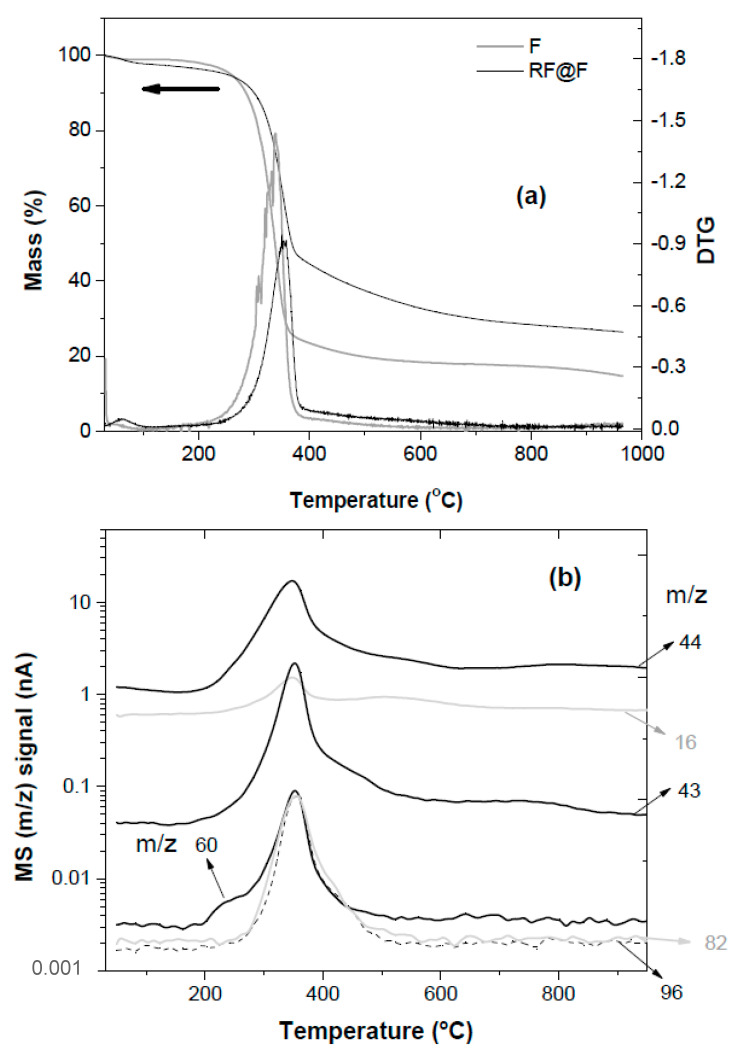
(**a**) Thermogravimetric (TGA and DTG) plots of cellulose fabric alone (F, grey line) and composite resin (RF@F, black line) during carbonization. (**b**) MS plots (from TGA/MS) of selected fragments produced during composite carbonization.

**Scheme 2:**
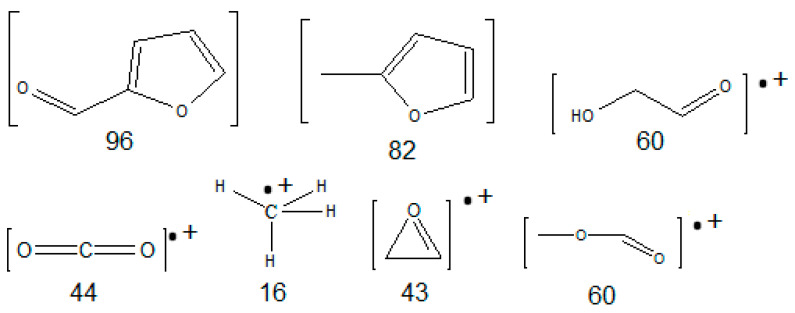
Structures of the fragments produced during RF-F carbonization.

The carbonization process of the composite is different from the fabric and RF alone (Figure 5a). The structural formula proposed for the *m*/*z* (radical cations = _•_+) values observed in the thermal decomposition of the composite are [92,93] (Scheme 2):

As can be seen, the main process occurs below 400 °C (Figure 5a), while some species (methane (16) and acetaldehyde (44)) are still produced at higher temperatures (400–600 °C). The furan-containing fragments (*m*/*z* = 82 and 96) (Figure 5b) are likely produced by the pyrolysis of the cellulosic fabric [94]. The signal of the masses *m*/*z*: 43 and 60 (<200 °C) are produced by the decomposition of the RF resin and detected at temperatures below 200 °C (Figure 5b). They are attributed to the loss of the methyl ether bridges between the benzene rings of the polymer during its curing process [95].

Further evidence of a different structure is shown during thermal oxidation (Figure S1, Supplementary Materials). RF_CTAB_ and RF burn in air, losing most of their mass. On the other hand, the combustion of RF@F reaches a finite mass value indicative of the formation of inorganic ash from the natural fiber. The hierarchical RF resin supported on fabric (RF@F) loses 50% of its mass at 470 °C, while the mesoporous RF, produced in the presence of CTAB, RF_CTAB_ reaches the same threshold at 520 °C. On the other hand, the non-porous RF xerogel losses 50% of its mass only at 580 °C. Therefore, the thermally driven reaction with oxygen follows the order hierarchical > mesoporous > non-porous.

#### 3.3.4. Electrochemical Properties

The composite materials could be used as electrode materials for electrochemical cells.

**Figure 6:**
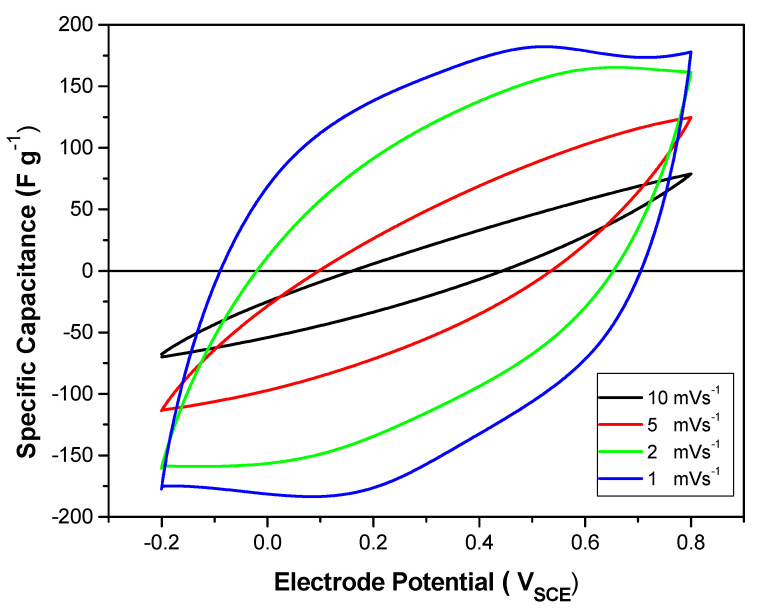
Cyclic voltammogram of a composite (c(RF@F)) electrode in 1 M H_2_SO_4_. Scan rate = see insert.

Small pieces of the material were tested in an aqueous solution (1 M H_2_SO_4_) in a conventional three-electrode cell. The cyclic voltammogram (Figure 6) shows a high double-layer capacitance with a broad wave around ca. 0.35 Vsce. The y-axis is expressed as specific capacitance relating to the scan rate and mass (see experimental part).

The wave is likely related to the oxidation/reduction of quinone functionalities present on the large carbon surface [96,97,98]. At low scan rates (1 mVs^−1^), maximum specific capacitances of up to 182 Fg^−1^ are measured. The mean specific capacitance in the whole potential window is ca. 150 Fg^−1^. As can be seen, the CV shape deforms with the scan rate since the current experimental increases (Figure S5, Supplementary Materials). Such CV can be simulated with a simple RC circuit response (Figure S6, Supplementary Materials). When the current is divided by the scan rate to obtain the capacitance, the shape of the CV at low scan rates approximates the square response of a purely capacitive (C) electrode, while the shape becomes closer to a purely resistive circuit (R, 45° line in the CV). Since the electrical conductivity of the composite is ca. 10.2 Scm^−1^, and the solution resistivity is below 10 ohms, the resistive component is related to the slow build-up of the double layer inside the porous structure.

**Figure 7:**
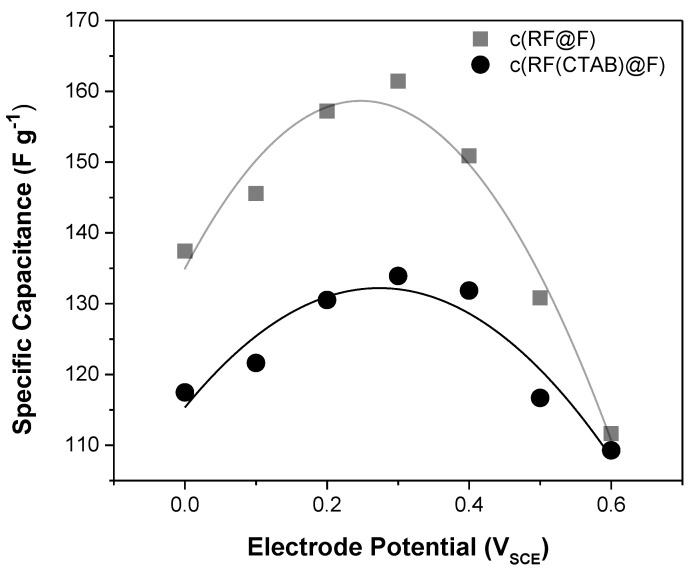
Specific capacitances of c(RF@F) (gray squares) and c(RF(CTAB)@F) (black circles) electrodes. The capacitances were measured from EIS at the low-frequency end (28 mHz) and different applied potentials in 1 M H_2_SO_4_ solution.

A way to measure specific capacitance without dynamic artifacts involves the use of electrochemical impedance spectroscopy (EIS). The Nyquist plot at different potentials (Figure S4, Supplementary Materials) show nearly constant impedance values (line parallel to the imaginary axis) at low frequency [99]. The specific capacitance (Sp_cap_) was at 2.8 mHz when the most surface area of the pores was accessed [100]. Also, it can be observed that the specific capacitance (Figure 7) depends on the electrode potential, showing a maximum value of ca. 160 Fg^−1^ at 0.25 Vsce. The shape of the Sp_cap_ vs. Electrode Potential curve is similar to that observed for c(RF_PDAMAC_) [44], with a maximum value at the mean redox potential of the quinone moieties present in the porous carbon surface.

It is noteworthy that covering the fabric with RF resin which contains a CTAB (RF_CTAB_@F), renders a carbon with a similar EIS response. However, similar curve but with a maximum specific capacitance of ca. 130 Fg^−1^ (ca. 20% lower) (Figure 7). The result suggests that the porosity stabilization by the fiber is not due to some soluble component remaining on the fabric.

**Figure 8:**
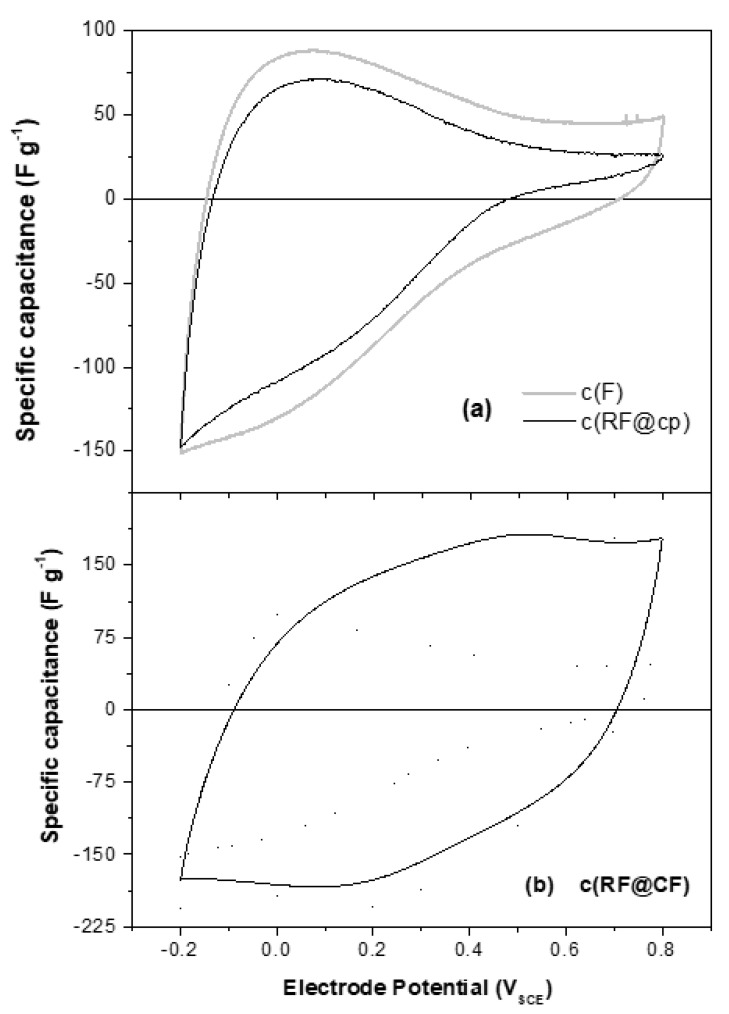
Cyclic voltammograms of (**a**) a carbonized RF resin deposited on a thin film of regenerated cellulose (c(RF@cp), black line) and a carbonized cellulose fiber fabric (c(F), grey line), and (**b**) a carbonized RF resin deposited onto carbonized cellulose fabric (c(RF@c(F)). Scan rate = 1 mVs^−1^. Solution = 1 M H_2_SO_4_.

An important question is which is the mechanism of templating or of the RF gel stabilization by the fabric. It is well known that carbonized cellulosic fibers could be activated to produce large surface areas that are used to store charge [101]. But the carbon fabric here has not been activated. Indeed, the CV of the carbonized fabric alone (Figure 8a) shows a different voltametric response which seems related to hydrogen storage in the micropores [102] and/or proton retention inside the micropores [103].

It could be envisaged that the hydrophilic chains of cellulose stabilize the gel upon drying and maintain the large porosity. To test that idea, RF resin was absorbed on a non-fibrous cellulose regenerated film (cellophane) and then carbonized. The carbon film shows a CV profile resembling the one obtained with the carbonized fabric alone (Figure 8a), suggesting that cellulose fibers can stabilize the porosity, but cellulose chains in a film do not produce the same effect.

Moreover, when a prepolymer resin of phenol-formaldehyde resin is adsorbed on the cellulosic fabric and then, carbonized, the electrochemical response of the composite carbon (c(PF@F), Figure S3, Supplementary Materials) shows a similar CV than those depicted in Figure 8a. It is known that phenol-formaldehyde forms a non-porous resin that renders compact glassy carbon [104]. Accordingly, the textural properties of c(F), c(RF@cp), and c(PF@F) show surface areas below 1 m^2^g^−1^.

However, it could be envisaged that both fibers and hydrophilic surfaces are necessary. There, the electrochemical properties of a carbon material are produced by the adsorption of RF resin on carbonized fabric, followed by carbonization of the composite c(RF@CF). The cyclic voltammetry (Figure 8b) shows a broad wave with a maximum specific capacitance of more than 150 Fg^−1^. This behavior is quite similar to that observed with carbonized RF resin adsorbed onto cellulosic fabric (c(RF@F)) (Figure 6, at a slow scan rate (1 mVs^−1^)). It seems that the hydrophilicity of the cellulose is not necessary to stabilize the porous RF gel since carbonized cellulose surfaces are quite hydrophobic. The results are significant because it suggests that other fibers could produce a templating or stabilizing effect similar to the fabric. Therefore, the stabilizing effect of RF gel porosity seems to be a physical effect due to gel (RF but not FF) adsorption on fibers.

A comparison with similar materials (Table 2) suggests that the specific capacitance of the material, while not the highest, is in the order of materials produced using cationic surfactants or cationic polyelectrolytes as stabilizers for the RF porosity during drying. Moreover, even nitrogen-doped and activated carbon materials [105], having ca. five times the surface area (2631 vs. 558 m^2^g^−1^), show a specific capacitance (227 Fg^−1^) in the same range (ca. 25% bigger) than that measured in c(RF@CF) (182 Fg^−1^).

However, the material shows better mechanical properties than fragile compact materials. No stabilizing additive [56] is necessary to produce the porous precursor resin. Moreover, no activation [105] or template removal [51] steps are required to produce the porous carbon.

Both c(RF@F) and c(RF_CTAB_@F) lose less than 6% specific capacitance after 1000 voltametric cycles (at 50 mVs^−1^).

An important question is the nature of ions used to compensate for the charge stored in the porous matrix. To shed light on the issue, we used Probe Beam Deflection techniques [106].

#### 3.3.5. Potential Driven Ion Exchange

The ion fluxes occur when the potential is changed relevant for applications, such as supercapacitors. An electrode that exchanges a single ion (e.g., protons) allows for building “rocking chair” supercapacitors [107], where the same ion travels from one electrode to the other during charge or discharge. In that way, a low volume of solvent is required to dissolve the electrolyte, improving the overall specific capacitance and power.

##### Acid Media

**Figure 9:**
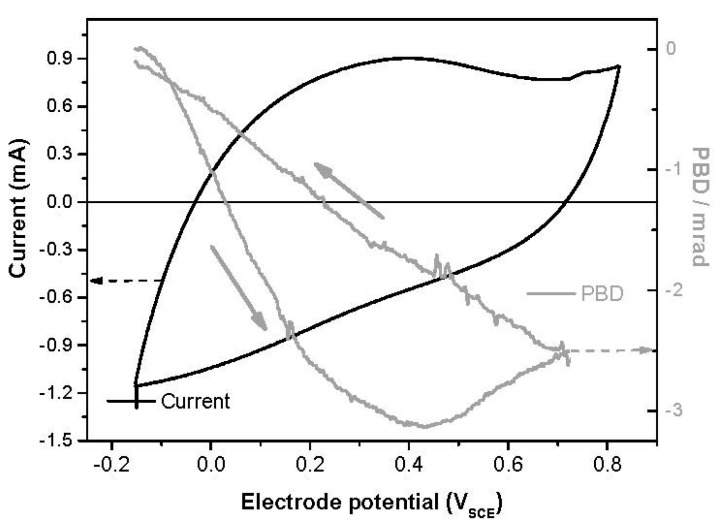
Cyclic voltammogram (black line) and deflectogram (grey line) of a composite mesoporous carbon in aqueous media (1 M H_2_SO_4_). Scan rate = 0.7 mVs^−1^. Dashed arrows point to the axis of the measurement. Thick full arrows show the sense of PBD signal change.

Using Probe Beam Deflection (PBD) [106], it is possible to ascertain the ion fluxes outside the carbon electrodes. The CV and PVD responses of a c(RF@F) electrode in acid are shown in Figure 9.

**Scheme 3:**
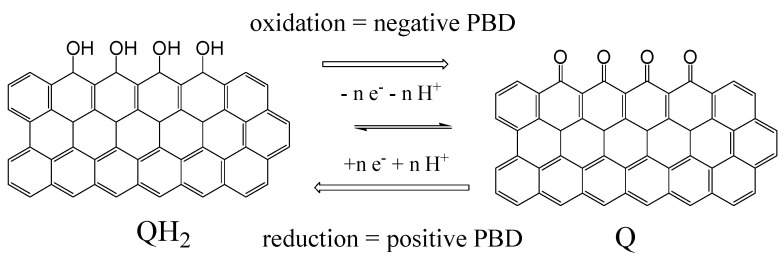
Surface reactions during oxidation/reduction of a c(RF@F) electrode.

The PBD signal (Figure 9, grey line) is negative during the anodic scan and positive during the cathodic scan. The sign of the signal agrees with a mechanism where ions are expulsed from the carbon during the anodic scan while ions are inserted in the cathodic scan. The PBD profile observed here is likely to be dominated by the pseudocapacitance, which is the oxidation/reduction of quinone-like functionalities on the carbon surface (Scheme 3):

The measured ion exchange is likely related to the proton release/intake and the oxidation/reduction of quinone moieties on the porous carbon surface (Scheme 2). This reaction has been observed in electrochemically oxidized glassy carbon, giving rise to a PBD profile similar to the one depicted in Figure 9 [108].

##### Neutral Media

**Figure 10:**
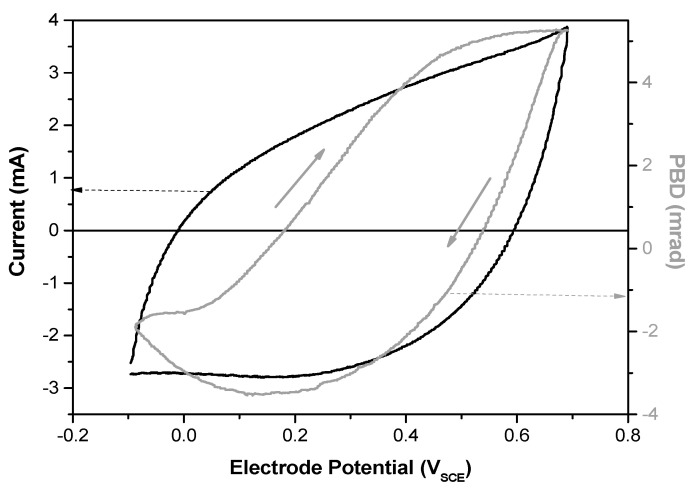
Cyclic voltammogram (CV, black line) and deflectogram (PBD, gray line) of a c(RF@F) electrode in 1 M KNO_3_. Scan rate = 1 mVs^−1^. Dashed arrows point to the axis of the measurement. Thick full arrows show the sense of PBD signal change.

A different behavior is observed when a neutral electrolyte (KNO_3_) is used. The CV does not show the wave related to hydroquinones/quinones redox reactions but a skewed double-layer response (see the simulation in Supplementary Materials, Figure S6). The deflectometry signal is positive during the anodic scan and negative during the cathodic scan (Figure 10).

This is indicative of ion insertion during the anodic scan and ion expulsion during the cathodic scan. Such behavior is related to the charging/discharging of the double layer at potentials positive to the potential of zero charge [99]. Moreover, a weak counter flux is observed at potentials below 0.1 Vsce (Figure 10). This suggests that the pzc is around 0.1 Vsce, and a counter flux is present at a potential more negative than the pzc. The PBD signal is similar to the one observed with c(RF_PDAMAC_) electrodes in the same electrolyte [52].

**Figure 11:**
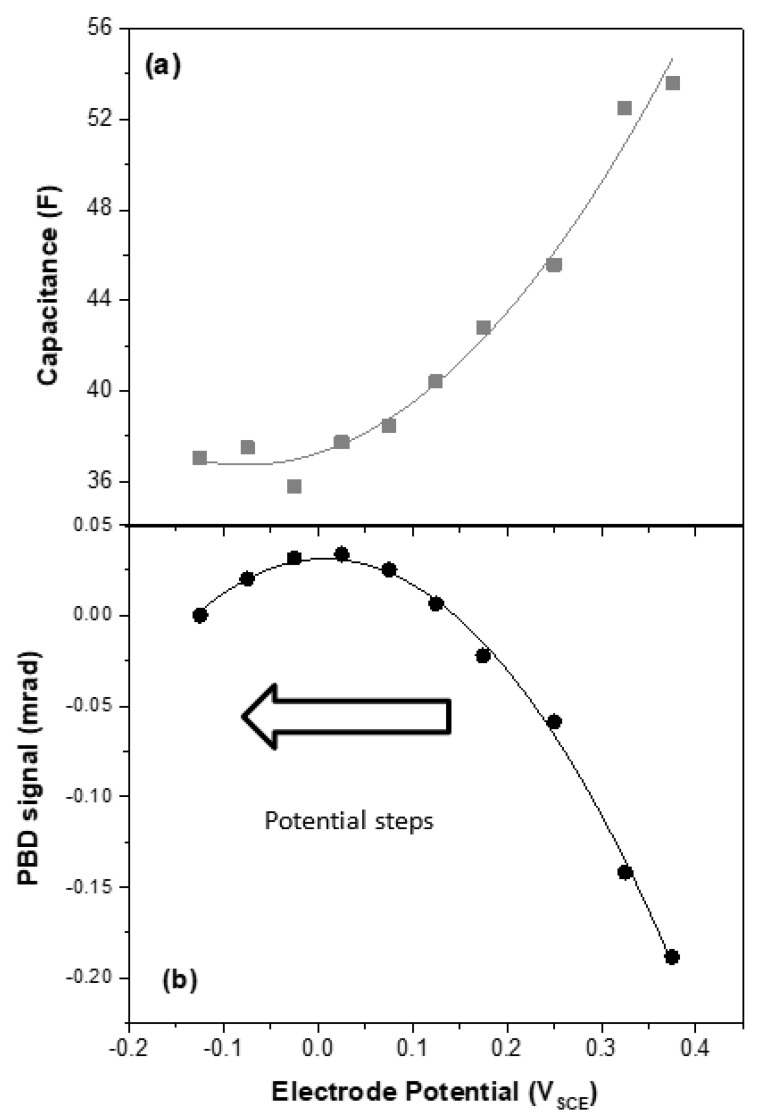
Simultaneous measurements of electrochemical capacitance (**a**, **gray squares**), measured by chronocoulometry, and ion population change (**b**, **black dots**), measured by chronodeflectometry, as a function of the potential. Electrode material = c(RF@F); in 1 M KNO_3_; potential step = −50 mV.

While cyclic voltadeflectometry gives qualitative information on the ion flux, a quantitative signal analysis requires digital simulation [109]. On the other hand, the chronodeflectometry (PBD signal monitored during the potential is stepped between two different potentials) has an analytical form for defined cases [110]. As can be seen in Figure S8 (Supplementary Materials), the chronodeflectometric data obtained with the c(RF@F) electrode in 1 M KNO_3_ could be fitted well with the profile of a discontinuous process [99]. That is, all ions by the electrode are exchanged during the initial period of the potential step because the film is diffusionally thin. This is reasonable as the hierarchical structure is made of a thin layer of mesoporous carbon supported by a macroporous carbon fiber fabric. Since the ion flux is monitored at a distance of the electrode/solution interface (>50 μm), the local concentration gradients of the fibers collapse into a planar diffusion gradient. The electrode potential is incremented by steps 50 mV, from 0.4 V to −0.15 V_SCE_, while the current and PBD signal is monitored. The charge exchanged during each pulse, ratioed by the potential step, gives the capacitance as a function of electrode potential (Figure 11a). A parabolic shape with a minimum at ca. 0.05 Vsce is found. The profile is similar to that observed for carbon aerogels [99] and agrees qualitatively with the potential dependence of charge predicted by the Gouy-Chapman-Stern model of double layer [111], with a potential of zero charge (pzc) of ca. 0.05 Vsce.

**Scheme 4:**
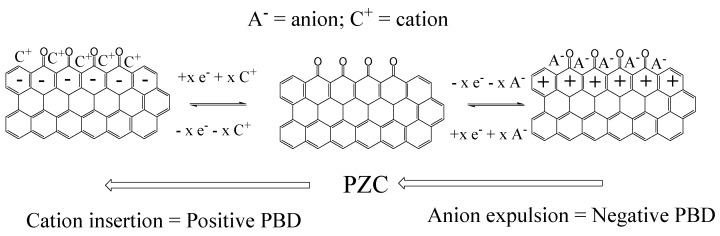
Ion exchange during charging/discharging of the double layer on the carbon surface.

From the chronodeflectometric data obtained during the same potential pulses (Figure S9, Supplementary Materials) it can be extracted the absolute PBD maxima which are related to the number of ions exchanged (Figure 11b). The changes in ion population as a function of electrode potential) shows a parabolic shape, with a maximum value at ca. 0.025 Vsce. At potentials negative to the pzc, cations (C^+^ = K^+^) are adsorbed from the double layer on the cathodic step, as shown by increasing values of PBD. At potentials positive to the pzc, anions (A^−^ = NO_3_^−^) are desorbed on the double layer, as shown by decreasing PBD values (Scheme 4).

The quinone groups remain unchanged since the first reduction potential of quinone moieties in neutral media is below −0.3 V_SCE_ [112]. Unlike the potential-driven ion exchange in acid, two different ions (anion and cation) are moving in a neutral (KNO_3_) solution. Therefore, it could not be used in “rocking chair” capacitors. However, the potential-driven ion exchange in neutral media could be applied in capacitive desalination devices [113] or ion-driven mechanical actuators [114].

## 4. Conclusions

It is possible to produce hierarchical (macro/meso/microporous) composite dry gels by adsorbing resorcinol-formaldehyde prepolymer on fiber fabrics. The presence of the fibers physically stabilizes the wet RF gel porous structure, avoiding pore collapse. The effect does not seem related to the chemical properties of cellulose since carbonized fibers also stabilize the porous RF gel during drying. Moreover, films of regenerated cellulose which do not contain fibers are unable to stabilize the RF gel and produce microporous gels.

The dried (in air) composite resin is pyrolyzed to give a carbon-carbon composite. The carbonization is monitored by TGA-MS, and volatile fragments produced from the cellulose and resin are detected. The presence of the fibers decreases the carbonization temperature.

The mechanical properties studied by nanoindentation are affected by the presence of the fiber matrix. Both the elastic modulus and hardness of the composite (c(RF@F)) are higher than those of a similar porous material (c(RF_CTAB_)) which does not contain fabric reinforcement. Therefore, the fiber-reinforced material has better mechanical properties than similar porous carbon.

The textural properties, measured by N_2_ adsorption/desorption isotherms, show a surface area of more than 550 m^2^g^−1^, with a significant portion of the surface areas as mesopores. The electrochemical properties were studied by cyclic voltammetry (CV), chronoamperometry/chronocoulometry (CA/CC), and electrochemical impedance spectroscopy (EIS). A maximum specific capacitance, in acid media, of 182 Fg^−1^ is measured by CV at a scan rate of 1 mVs^−1^, while a maximum capacitance of 160 F g^−1^ is measured by EIS at 2.8 mHz.

The effect of potential on the ion exchange was measured using the Probe Beam Deflection technique. In acid media, ion expulsion is observed in anodic scans, with ion insertion in the cathodic scan. The ion fluxes seem dominated by the proton exchange of the quinone moieties (pseudocapacitance) present in the porous surface. On the other hand, PBD signals due to two different ion fluxes are observed in neutral media. At electrode potentials more positive than a threshold potential (ca. 0.05 V_SCE_), ions are expelled during cathodic steps, while at potentials more negative, ions are inserted in the porous carbon during cathodic pulses. The capacitance shows a parabolic dependence with the potential of zero charge (pzc). The ion population change, measured by chronodeflectiometry, shows a maximum at the pzc. The observed potential-driven ion exchange supports their use in desalination devices or ion-based electromechanical actuators.

## Figures and Tables

**Table 1:** Elastic modulus and hardness of porous carbon materials.

Material	Elastic Modulus (GPa)	Hardness (GPa)
Hierarchical carbon: c(RF@F)	14.2 ± 2.03	2.21 ± 0.34
Mesoporous carbon: c(RF_CTAB_)	6.6 ± 0.34	0.93 ± 0.07

**Table 2:** Comparison of surface areas and specific capacitance of mesoporous carbon materials.

Carbon Material	Surface Area (m^2^ g^−1^)	Specific Capacitance (CV, F g^−1^) *	Specific Capacitance (EIS, F g^−1^) ^#^	Reference
c(RF stab. CTAB)	536	154	194	[56]
c(RF stab. CTAB)	671	145	145	[47]
c(RF stab. PDAMAC)	725	210	--	[52]
c(RF stab PDAMAC)	675	97	142	[53]
c(RF stab. CTAC)	453	135	--	[44]
c(RF stab. BzACl)	535	--	179	[48]
c(RF stab CTAB + NiO)	637	271	216	[51]
c(chitin) activated (NaOH)	2631	227 ^&^	--	[105]
c(RF stab. Cel. Fibers)	558	182	160	This work
c(RF stab CTAB + Cel. Fibers)	--	130	--	This work

* cyclic voltammetry at 1 mVs^−1^ (integral value). in 1 M H_2_SO_4_. ^#^ Electrochemical impedance spectroscopy at 2.8 mHz. ^&^ measured at 20 mVs^−1^.

## Supplementary Materials

The following supporting information can be downloaded at: https://www.mdpi.com/article/10.3390/ma16052101/s1, Experimental details (PBD set-up), Figure S1: TGA during oxidation, Figure S2: CV of c(RFCTAB), Figure S3: CV of (c(PF@F)), Figure S4: Nyquist plots (EIS) of c(RF@F), Figure S5: experimental CV (current-E) of c(RF@F), Figure S6: simulated CV (i-E) of a RC circuit, Figure S7: simulated CV (Scap-E) of a RC circuit, Figure S8: chronodeflectometry of c(RF@F) in neutral mdia, Figure S9: Chronodeflectometry of c(RF@F) in neutral media during potential pulses (50 mV), varying the initial potential.
