# Supplementary material for: Hierarchical Biobased Macroporous/Mesoporous Carbon: Fabrication, Characterization and Electrochemical/Ion Exchange Properties
Source: Materials (Basel). 2023 Mar 5;16(5):2101. doi: 10.3390/ma16052101 (PMC10004673; doi:10.3390/ma16052101)
Supplement: Supplementary file 1 [file materials-16-02101-s001.zip › materials-2217140-supplementary.pdf]

Experimental details

Probe Beam deflection set-up

The basic components of the PBD system are a 5 mW He-Ne laser (Melles Griot, 05 LHP11) and a bicell position sensitive detector (UDT PIN SPOT/2D). The laser beam is focused by a 50 mm lens to a diameter of roughly 60 ◎m in front of the planar electrode. The electrochemical cell, an optical glass cuvette with 2 x 2 x 4 cm dimensions (1 cm of path length), is mounted on a 3 axis tilt table (Newport). A micrometric translation stage allows for controlled positioning of the sample with respect to the laser beam in 10 ◎m steps. The position sensitive detector is placed 25 cm behind the electrochemical cell and has a sensitivity of 3 mV/1 μm, which resulted in a deflection sensitivity of 1 mrad/V. All parts of the system are fixed on an optical rail and the whole set-up is mounted on an optic table (Melles Griot). The deflection signal was processed using a position monitor (UDT 201 DIV). Due to the fact that PBD signal have to be monitored at long times (> 50 s), the whole system was warmed up for 24 Hs before each measurement to eliminate thermal fluctuations. The signal of the two photodiodes making the bicell detector were subtracted and normalized to the overall signal to eliminate laser intensity fluctuations. The glass cell contains a counter electrode of aerogel (with geometrical area 5 times the working electrode) and a Ag/AgCl (3 M NaCl) miniature reference electrode (BAS) separated from the solution with a Vycor diaphragm. The working electrodes were carbon composites plates plates (width 2 mm) attached onto Teflon plates with sides and back sealed and the active side unpolished. The electrochemical control of Probe Beam Deflection experiments was performed using a potentiostat (AMEL 2049). The set-up was controlled by a PC through a LabPC AD/DA card running in homemade software performed with LabView (National Instruments). The deflection and the electrochemical data were stored in the PC.

FIGURES

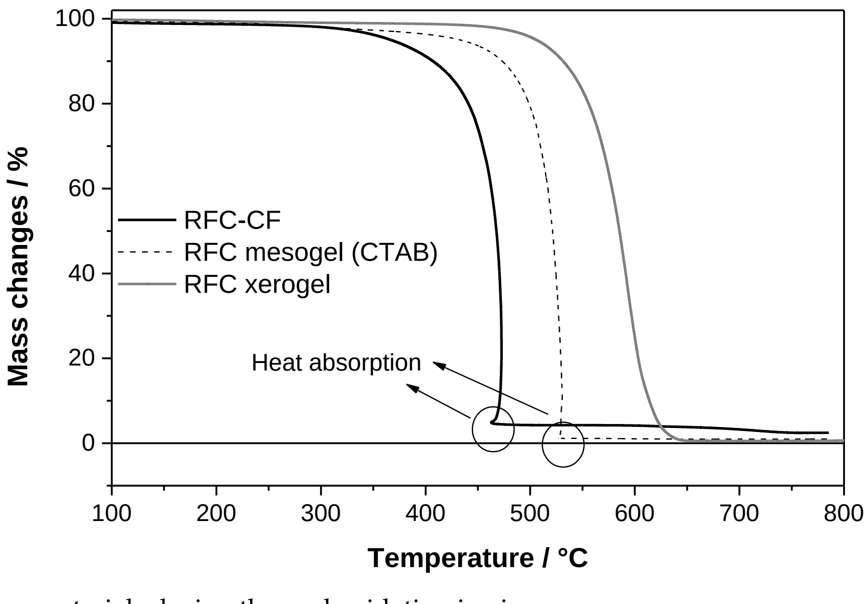

Figure S1 TGA plots of different carbon materials during thermal oxidation in air.

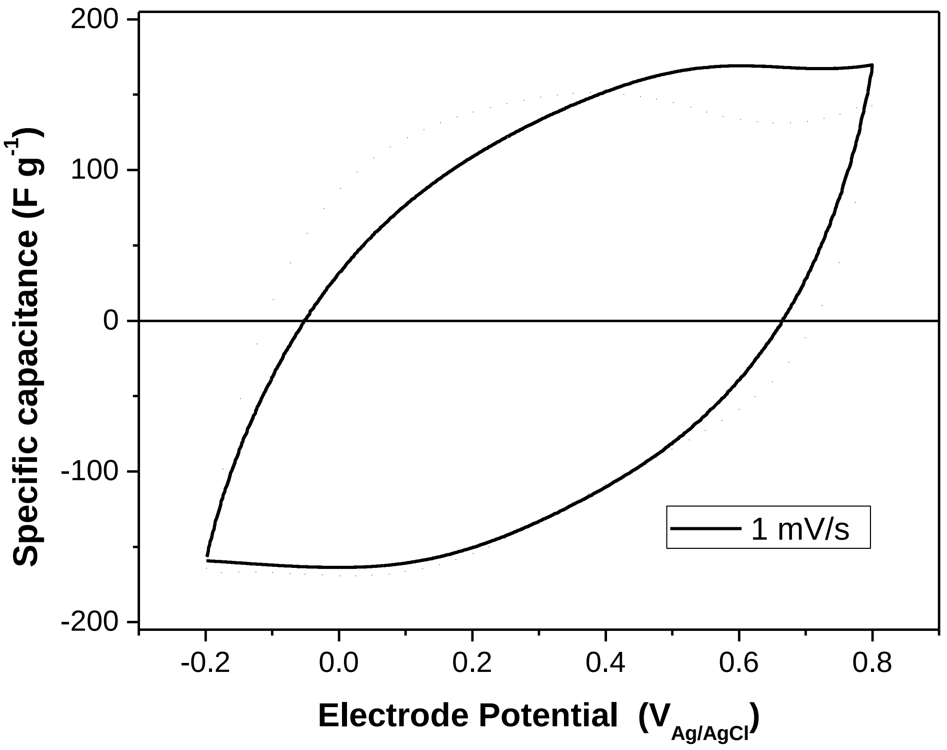

Figure S2 . cyclic voltammogram of a carbonized composite made of resorcinol-formaldehyde resin (RF) with a surfactant (CTAB) adsorbed onto a cellulosic fibre fabric c(RF(CTAB)@F) electrode in 1 M H_2SO_4

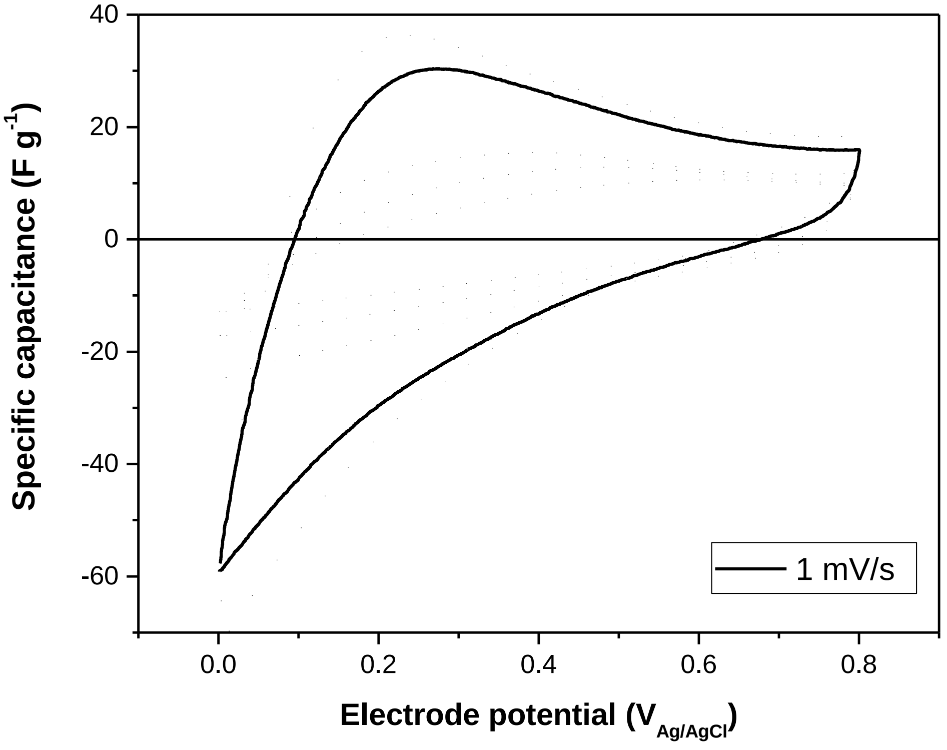

Figure S3. cyclic voltammogram of a carbonized composite made of phenol-formaldehyde resin adsorbed onto a cellulosic fibre fabric (c(PF@F)). Scan rate = 1 mV/s. Solution = 1 M H_2SO_4.

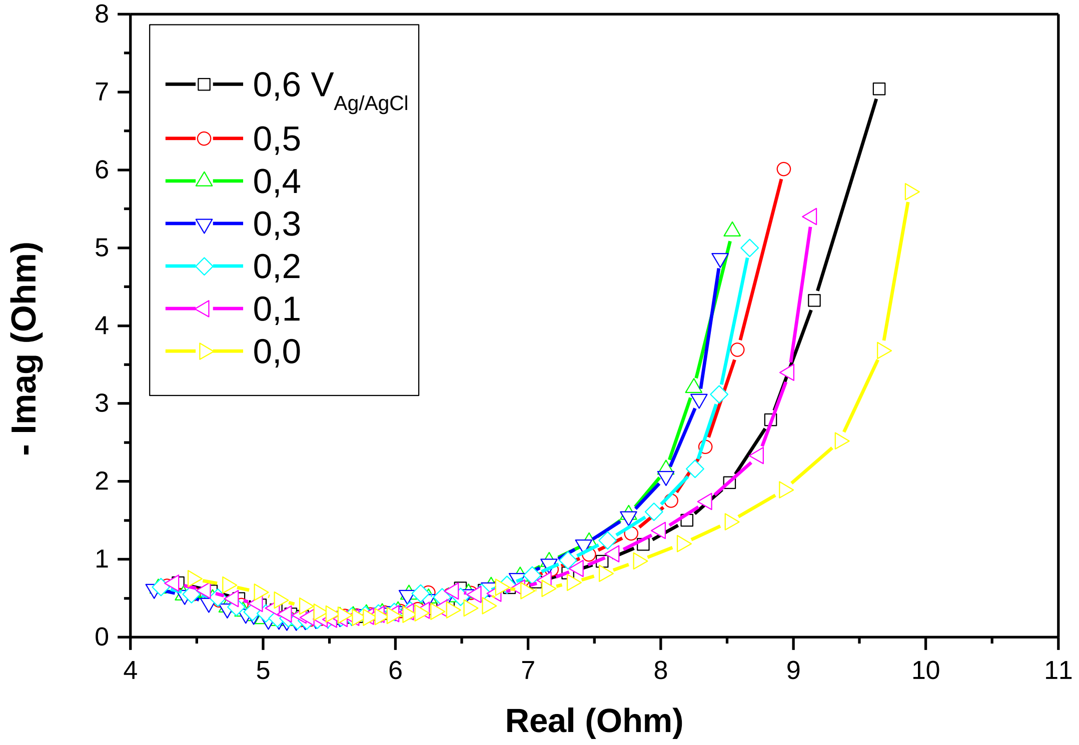

Figure S4: Complex plane plots (Nyquist) of the AC impedance data taken at different frequencies (50 kHz to 3 mHz). Solution = 1 M H_2SO_4.

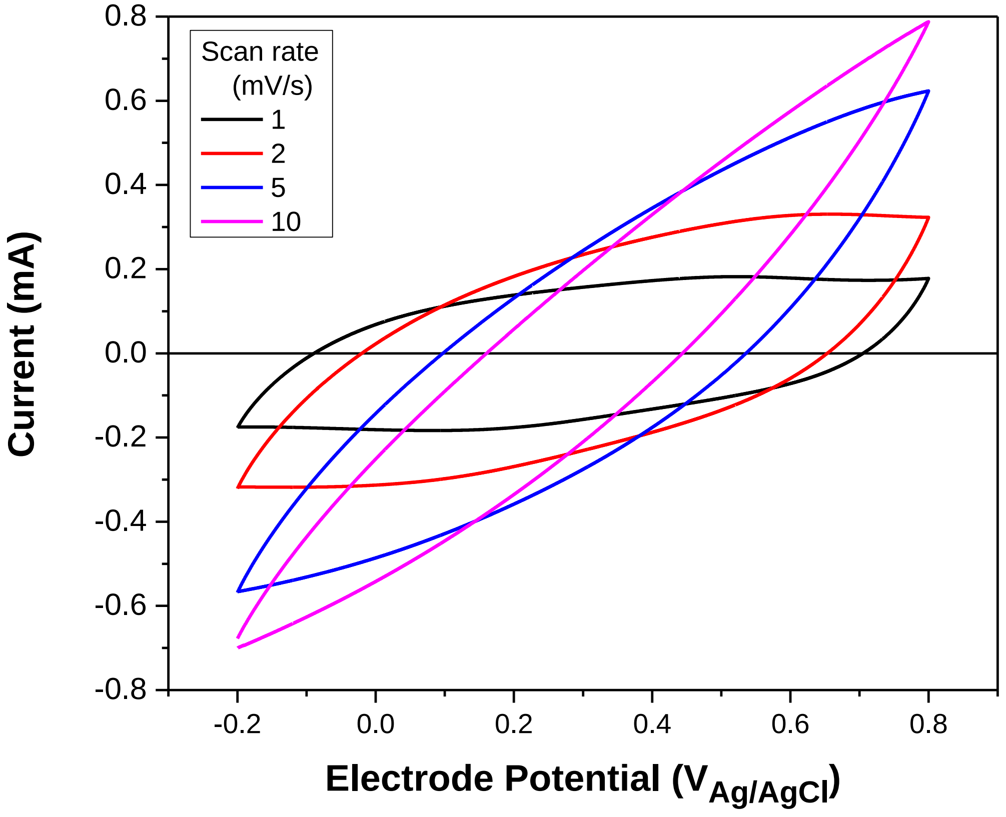

Figure S5. Experimental CV of c(RF@F) in 1 M H_2SO_4.

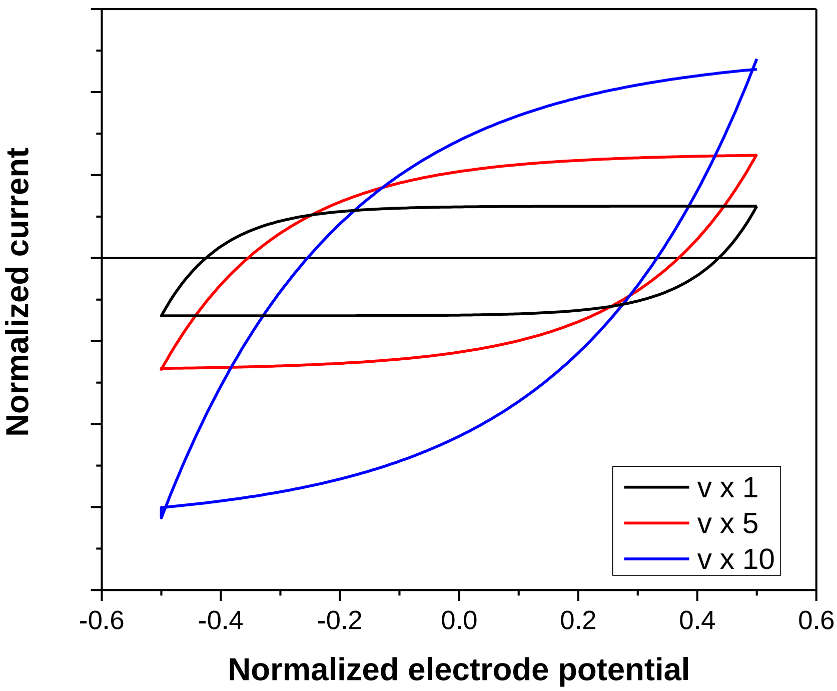

Figure S6. Simulated CV (i-E) at different scan rates with a simple RC circuit

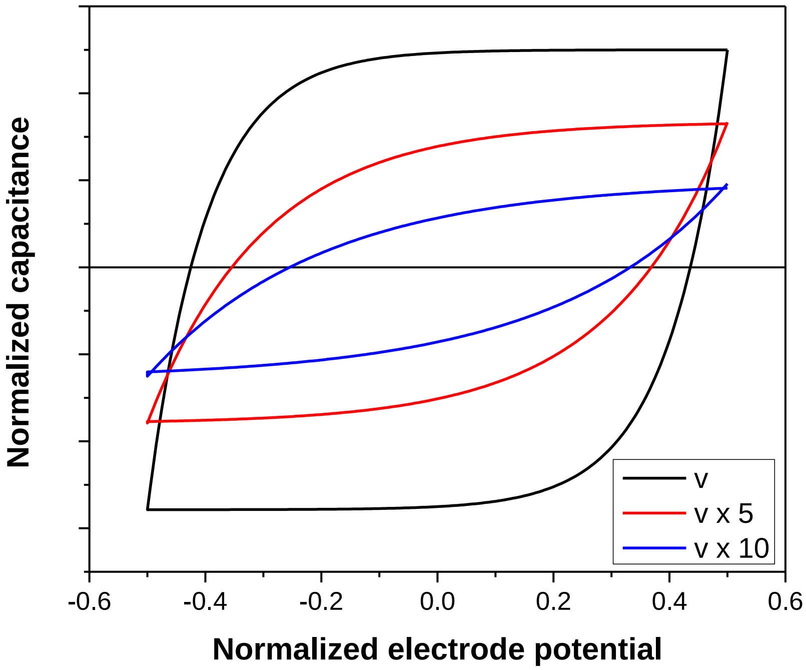

Figure S7. Simulated CV (C-E) at different scan rates with a simple RC circuit

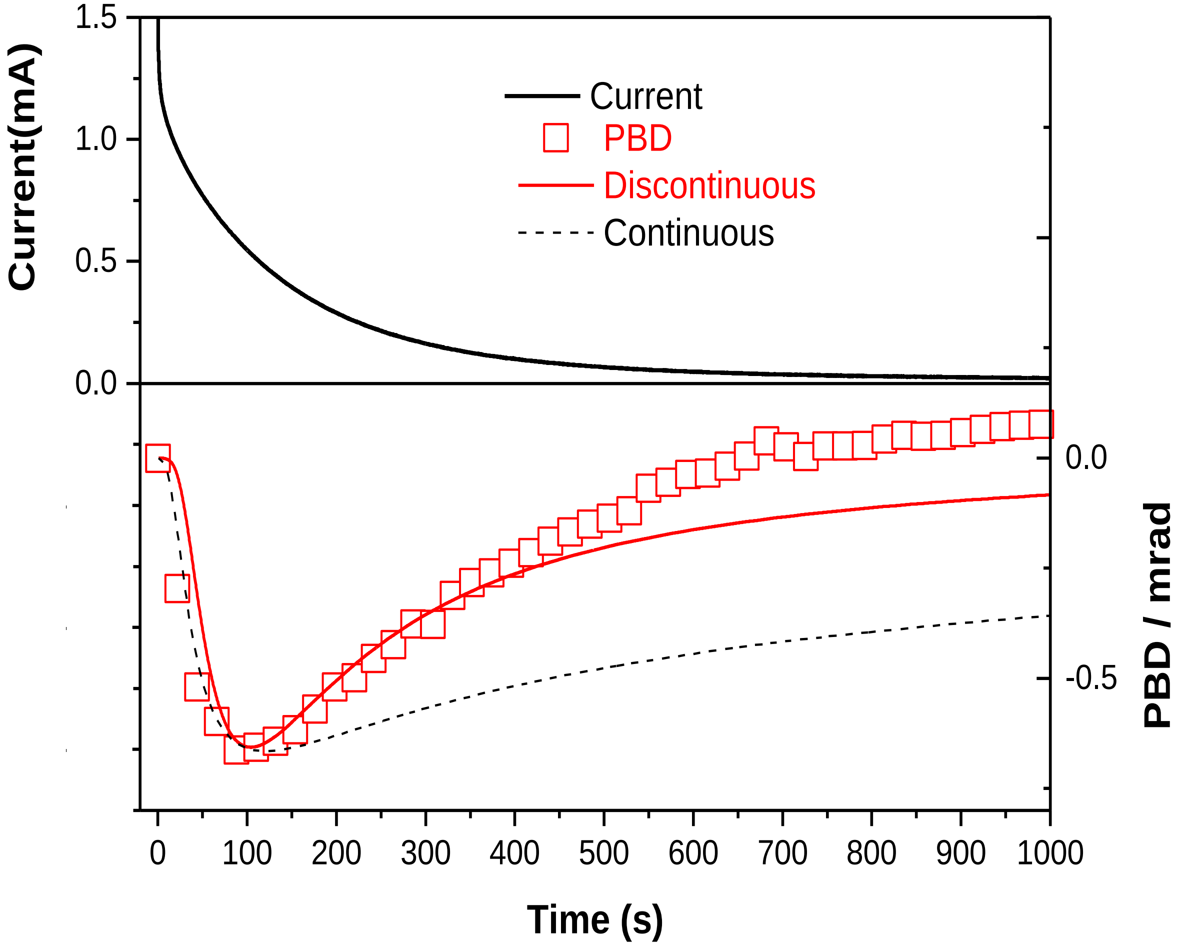

Figure S8 Chonoamperometric (upper panel) and chronodeflectometric (down panel) response of a c(RF@F) electrode in aqueous media (0.5 M KNO_3). The potential of the electrode is switched between 0.3 and 0.25 Vsce. The grey line is simulated using a discontinuous process.

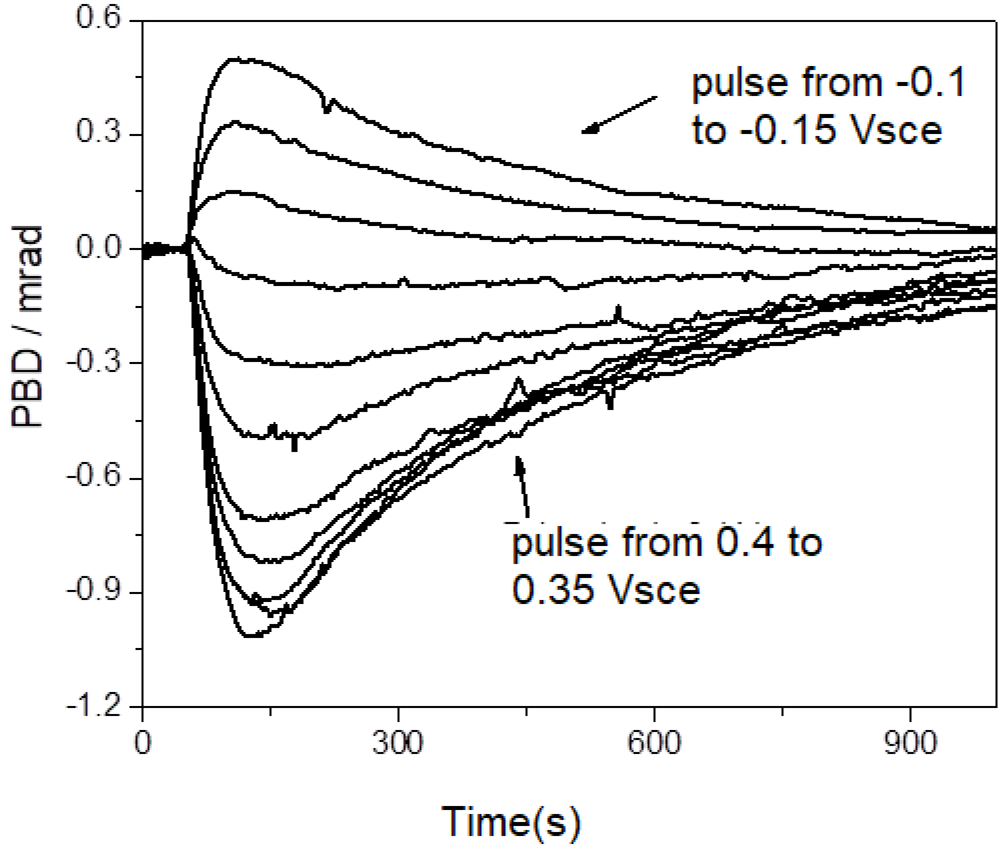

Figure S9 Chronodeflectometric responses of a c(RF@F) electrode in aqueous media (0.5 M KNO_3) during potential pulses (50 mV) at different initial potentials. The initial potential of the electrode is changed between 0.4 and -0.15 Vsce.
